# A critical role of the K_Ca_3.1 channel in mechanical stretch‐induced proliferation of rat bone marrow‐derived mesenchymal stem cells

**DOI:** 10.1111/jcmm.15014

**Published:** 2020-02-17

**Authors:** Xiaoling Jia, Hao Su, Xinlan Chen, Yangbi Huang, Yufan Zheng, Pei Ji, Chao Gao, Xianghui Gong, Yan Huang, Lin‐Hua Jiang, Yubo Fan

**Affiliations:** ^1^ Key Laboratory for Biomechanics and Mechanobiology of Ministry of Education School of Biological Science and Medical Engineering Beihang University Beijing China; ^2^ Beijing Advanced Innovation Centre for Biomedical Engineering Beihang University Beijing China; ^3^ School of Biomedical Science Faculty of Biological Sciences University of Leeds Leeds UK; ^4^ Department of Physiology and Neurobiology Xinxiang Medical University Xinxiang China; ^5^ Beijing Key Laboratory of Rehabilitation Technical Aids for Old‐Age Disability National Research Center for Rehabilitation Technical Aids Beijing China

**Keywords:** bone marrow‐derived mesenchymal stem cells, cell proliferation, K_Ca_3.1 channel, mechanical stretch

## Abstract

Mechanical stimulation is an important factor regulating mesenchymal stem cell (MSC) functions such as proliferation. The Ca^2+^‐activated K^+^ channel, K_Ca_3.1, is critically engaged in MSC proliferation but its role in mechanical regulation of MSC proliferation remains unknown. Here, we examined the K_Ca_3.1 channel expression and its role in rat bone marrow‐derived MSC (BMSC) proliferation in response to mechanical stretch. Application of mechanical stretch stimulated BMSC proliferation via promoting cell cycle progression. Such mechanical stimulation up‐regulated the K_Ca_3.1 channel expression and pharmacological or genetic inhibition of the K_Ca_3.1 channel strongly suppressed stretch‐induced increase in cell proliferation and cell cycle progression. These results support that the K_Ca_3.1 channel plays an important role in transducing mechanical forces to MSC proliferation. Our finding provides new mechanistic insights into how mechanical stimuli regulate MSC proliferation and also a viable bioengineering approach to improve MSC proliferation.

## 1 INTRODUCTION

Mesenchymal stem cells (MSCs) have many promising applications in regenerative medicine.^1^, ^2^ The capability of MSC proliferation however declines upon in vitro expansion.^3^, ^4^ Identification of practical methods to maintain or increase MSC proliferation to increase their availability is helpful to their clinical applications. Compelling evidence shows that mechanical stimulation is an important factor regulating MSC functions, including proliferation, but the underlying mechanism is far from understood.^5^, ^6^, ^7^ K_Ca_3.1 channel, also known as KCNN4, IK_Ca_ and SK4, is an intermediate‐conductance member of the Ca^2+^‐activated K^+^ channel family and is widely expressed in both excitable and non‐excitable cells, where it plays an important role in various cell functions.^8^ Interestingly, there is increasing evidence to support the K_Ca_3.1 channel is engaged in MSC proliferation.^9^, ^10^, ^11^ Previous studies showed that the expression of the K_Ca_3.1 channel in human umbilical vein endothelium cells was up‐regulated by fluid flow‐induced shear stress, or its channel activity in vascular smooth muscle cells was enhanced by hypotonic solution‐induced membrane stretch.^12^, ^13^ These findings led us to hypothesize a role of the K_Ca_3.1 channel in mechanical stretch‐induced regulation of MSC proliferation. Here, we showed that application of mechanical stretch to rat bone marrow‐derived MSCs (BMSCs) significantly increased cell proliferation, mainly via altering cell cycle progression. Such mechanical stretch also up‐regulated the K_Ca_3.1 expression at mRNA, protein and functional levels. Importantly, mechanical stretch‐induced stimulation of BMSC proliferation and alteration in cell cycle progression were prevented by pharmacological inhibition of the K_Ca_3.1 channel or siRNA‐mediated knockdown of the K_Ca_3.1 expression. Taken together, our results provide compelling evidence to support an important role of the K_Ca_3.1 channel in mechanical stimuli‐induced stimulation of MSC proliferation, thus revealing a new molecular mechanism in transducing mechanical forces to regulate MSC proliferation and identifying a viable bioengineering approach to improve MSC proliferation.

## 2 MATERIALS AND METHODS

### 2.1 Cell isolation, culture and siRNA transfection

Isolation of BMSCs from 30‐day‐old male Sprague‐Dawley rats, BMSC characterization and culture were previously described.^14^ All experiments were approved by the Animal Research Ethics Committee of Beihang University. Passage 3‐5 BMSCs were used. Transfection with K_Ca_3.1‐specific siRNA (siKCa3.1) or control siRNA (siCTL) was described previously,^15^ and the efficiency of knockdown was confirmed by Western blotting (Figure S1).

### 2.2 Application of mechanical stretch

Cells were seeded at density of 1 × 10^5^ on silicone chambers pre‐coated with collagen I (Becton Dickinson). When cells reached 80% confluence, the silicone chambers were mounted to a stretch device (STREX, Japan) and were exposed to stretch by 2.5%, 5%, 10% and 15% for 6, 12 and 24 hours. Cells cultured without stretch were used as static control (SC). To block the K_Ca_3.1 channel, 100 nmol/L TRAM‐34 (Alomone) was added into the culture medium.

### 2.3 Reverse transcription‐polymerase chain reaction (RT‐PCR)

Total RNA extraction and RT‐PCR were described in the Supplementary File. The primers used were listed in Table S1.

### 2.4 Protein expression determination

The cell surface K_Ca_3.1 protein expression was determined using fluorescence‐activated cell sorting (FACS) as previously described.^16^ Cells were fixed without permeabilization and incubated with FITC‐conjugated antibody recognizing the extracellular domain of IK_Ca_3.1 (Alomone). Isotype control IgG was used as control. The fluorescence intensity was determined by FACSCalibur (Becton Dickinson) and analysed using CellQuest software.

### 2.5 Cell proliferation

Cell proliferation was examined by determining the number of living cells using CCK‐8 kits according to the manufacturer's instructions (Dojindo). The absorbance at 450 nm was measured using a microplate reader (Thermo Scientific).

### 2.6 Cell cycle analysis

Cells, after being synchronized for 24 hours in serum‐free medium, were subjected to mechanical stretch for further 24 hours in media containing 10% foetal bovine serum. The cell cycle distribution was determined by FACSCalibur (Becton Dickinson) and analysed by ModFit software as previously described.^16^

### 2.7 Electrophysiology

Whole‐cell patch‐clamp recording of the K^+^ currents was performed using a HEKA amplifier (Lambrecht) as described previously^9^ and detailed in the Supplementary File. Membrane stretch was induced by applying hypotonic solution as described previously.^13^ The K_Ca_3.1 channel currents were derived from TRAM‐34‐sensitive current components.

### 2.8 Statistical analysis

Data are presented as mean ± standard deviation, where appropriate. Statistical analysis was conducted using Student's test or one‐way ANOVA and *post hoc* Fisher's test as indicated. *P* < .05 was considered statistically significant.

## 3 RESULTS

**Figure 1:**
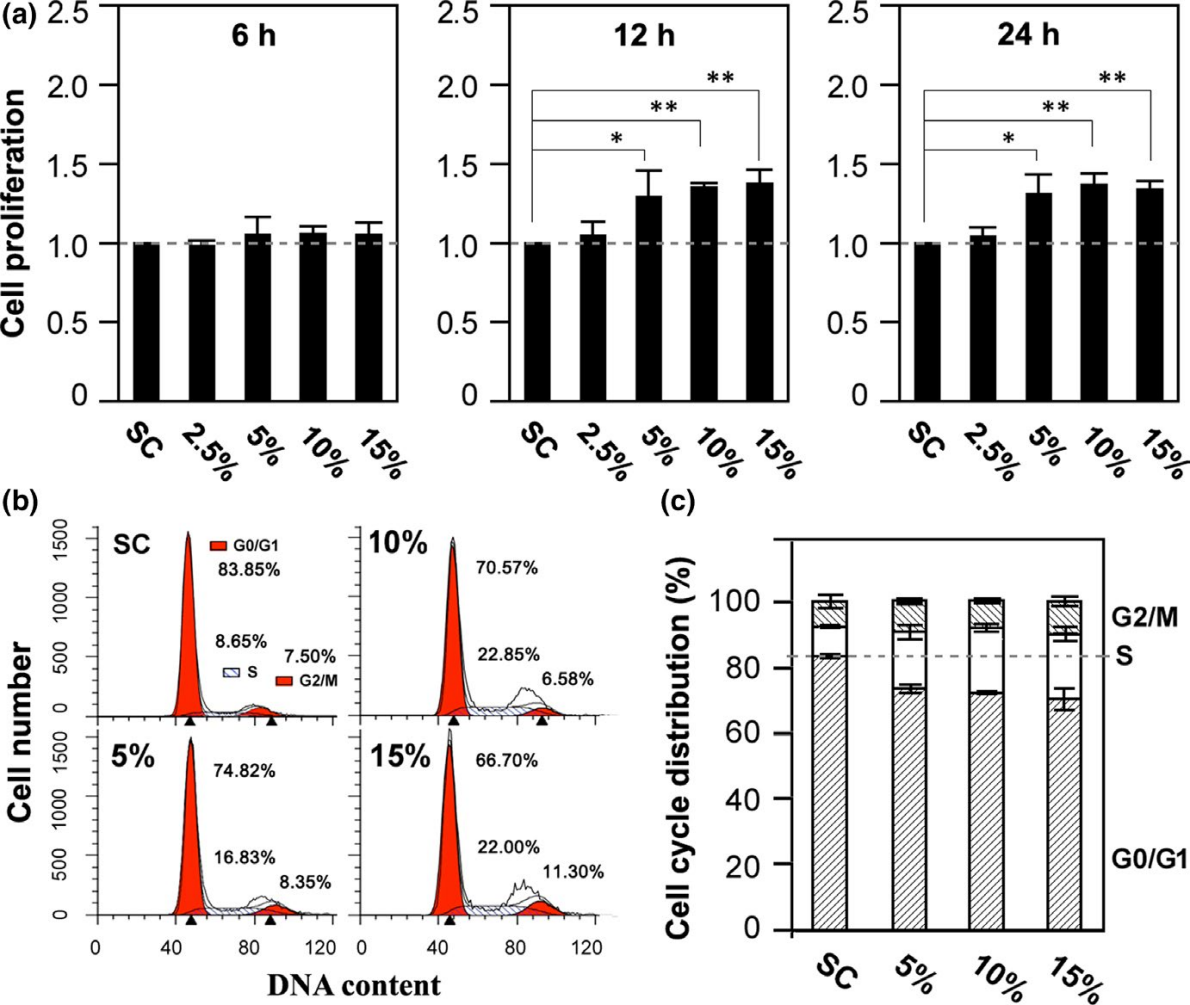
Effects of mechanical stretch on BMSC proliferation. A, summary of the effects of exposing BMSC to 2.5%‐15% mechanical stretch for 6, 12 and 24 h on cell proliferation relative to static control (SC). The mean data are from five independent experiments. *P < .05 and **P < .01 compared to SC using one‐way ANOVA and *post hoc* Fisher's test. B‐C, representative analysis of cell cycle distribution in cells under indicated conditions (B), and summary of the mean data from 4 independent experiments (C). Cells were fixed with 70% ethanol overnight, incubated in PBS staining solution (20 μg/mL propidium iodide, 100 μg/mL RNase A, and 0.1% Triton X‐100) at 37°C for 30 min and analysed by FACS on FL‐2 channel. The data were analysed using ModFit software

We started with examining the effects of mechanical stretch on BMSC proliferation. We chose mechanical stretch from 2.5% to 15%, the most commonly used range in study of mechanical stretch‐induced regulation of MSC functions.^5^, ^6^, ^7^, ^17^, ^18^ As shown in Figure 1A, exposure to mechanical stretch for 6 hours, regardless of mechanical stretch strength, had no effect, and prolonged exposure for 12 and 24 hours of 5%, 10% and 15%, but not 2.5%, resulted in a significant increase in living cell number (Figure 1A). Taken together, these results show that mechanical stretch stimulates BMSC proliferation in a time‐ and strength‐dependent manner. To further understand how mechanical stretch accelerated BMSC proliferation, we analysed cells in different phases of the cell cycle after exposure to 5%‐15% stretch for 24 hours (Figure 1B‐C). Exposure to mechanical stretch increased the percentage of cells in the S phase and reduced the percentage of cells in the G0/G1 phase (Figure 1C), suggesting that mechanical stretch stimulates cell proliferation via promoting cell cycle progression.

**Figure 2:**
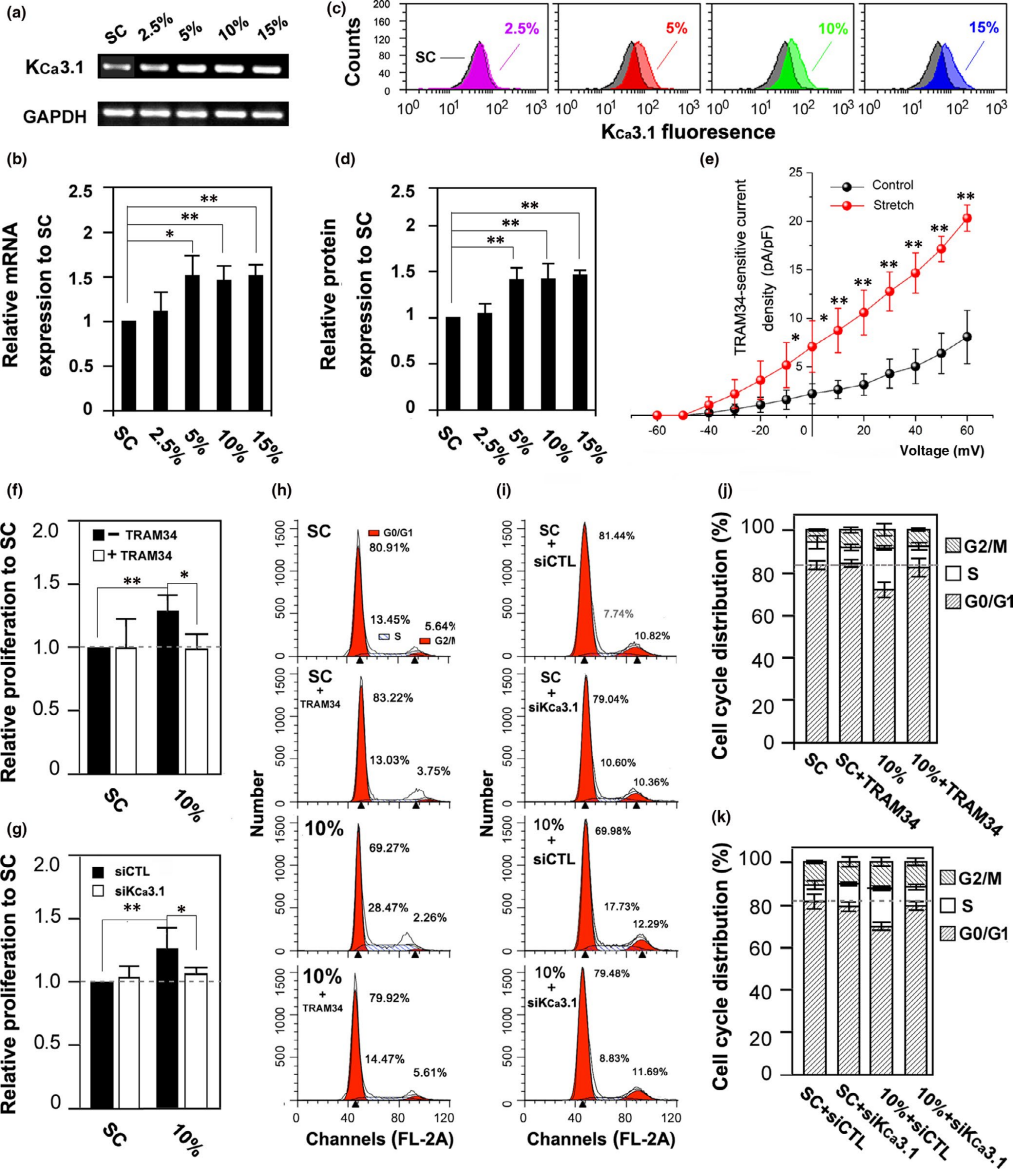
Effects of mechanical stretch on K_Ca_3.1 expression and activity and the role of K_Ca_3.1 channel in mechanical stimulation of BMSC proliferation. A‐D, effects of exposing BMSC to 2.5%‐15% mechanical stretch for 24 h on the K_Ca_3.1 expression levels. A and C, representative results showing the K_Ca_3.1 mRNA expression using RT‐PCR and K_Ca_3.1 cell surface protein expression using flow cytometry. B and D, summary of the mean data as shown in (A) and (C), respectively, from six independent experiments. *P < .05 and **P < .01, using one‐way ANOVA and *post hoc* Fisher's test. E, summary of the I‐V relationship curves of the mean TRAM‐34 sensitive K^+^ current densities recorded from seven cells for each condition. Control, isotonic solution; Stretch, hypotonic solution. *P < .05 and **P < .01. Student's *t* test was used to compare the current density between control and stretch at the same potential. F‐K, summary of BMSC proliferation and cell cycle under indicated conditions after treatment with 100 nmol/L TRAM34 (F, H, J) or siRNA‐mediated knockdown of the K_Ca_3.1 expression (G, I, K), from four independent experiments. *P < .05 and **P < .01, using one‐way ANOVA and *post hoc* Fisher's test

As introduced above, the K_Ca_3.1 channel is critically engaged in MSC proliferation.^9^, ^10^, ^11^ We were therefore interested in the effects of mechanical stretch on the K_Ca_3.1 channel expression. Exposure to mechanical stretch of 5%‐15%, but not 2.5%, for 24 hours increased the expression of K_Ca_3.1 at the mRNA level shown by RT‐PCR (Figure 2A‐B) and also at the protein level shown by FACS (Figure 2C‐D). In addition, whole‐cell recording showed that the amplitude of TRAM34‐sensitive K^+^ currents was enhanced by membrane stretch induced using hypotonic solution (Figure 2E). Taken together, these results indicate that mechanical stimulation significantly enhances the K_Ca_3.1 channel expression and activity. We finally investigated whether the K_Ca_3.1 channel plays a role in mechanical stretch‐induced stimulation of BMSC proliferation. Treatment with TRAM34, a K_Ca_3.1 channel‐specific inhibitor, prevented mechanical stretch‐induced increase in cell proliferation, without effect on cell proliferation under normal control condition (Figure 2F). Similarly, siRNA‐mediated knockdown of the K_Ca_3.1 expression (Figure S1) suppressed mechanical stretch‐induced stimulation of cell proliferation (Figure 2G). Analysis of cell cycle further revealed that pharmacological inhibition of the K_Ca_3.1 channel or genetic depletion of the K_Ca_3.1 expression prohibited mechanical stretch‐induced arrest of cell cycle in the G0/G1 phase (Figure 2H‐K). Collectively, these results consistently support a critical role of the K_Ca_3.1 channel in mediating mechanical stretch‐induced stimulation of BMSC proliferation.

## 4 DISCUSSION

We here show that exposure to mechanical stretch stimulates BMSC proliferation (Figure 1A), in support of the notion that mechanical force regulates MSC proliferation.^5^, ^6^, ^7^ We further revealed mechanical stretch‐induced stimulation of BMSC proliferation via promoting cell cycle progression (Figure 1B‐C). Moreover, extended exposure to mechanical stretch strongly up‐regulated the K_Ca_3.1 expression in BMSC (Figure 2A‐D) and, interestingly, acute exposure to hypotonic solution enhanced the K_Ca_3.1 channel activity (Figure 2E). More importantly, inhibition of the K_Ca_3.1 channel with TRAM‐34 (Figure 2F) or siRNA‐mediated knockdown of the K_Ca_3.1 expression (Figure 2G) strongly suppressed mechanical stretch‐induced BMSC proliferation, and such pharmacological or genetic intervention of the K_Ca_3.1 channel inhibited mechanical stretch‐induced alteration in cell cycle (Figure 2H‐K). Previous studies using mouse and dog BMSCs reported engagement of the K_Ca_3.1 channel in cell proliferation.^9^, ^11^ However, under our static control condition, inhibition of the K_Ca_3.1 channel in rat BMSC had no effect on cell proliferation (Figure 2F). It is noted that the expression level of Ca^2+^‐activated K^+^ channels, including K_Ca_3.1, in BMSCs is strongly species‐dependent,^19^ which may contribute to the different observations in the present and previous studies, but the exact reason remains unknown. There is evidence that mechanical stimulation induces intracellular Ca^2+^ increase and cell proliferation in BMSCs.[19, 20, 21] This is consistent with our finding that mechanical stimulation via exposing to hypotonic solution enhanced the K_Ca_3.1 channel activity (Figure 2E). Thus, it is tempting to hypothesize that the K_Ca_3.1 channel plays a critical role in coupling mechanical stimulus‐induced Ca^2+^ signal to activation of the downstream signalling pathways to stimulate MSC proliferation. It has been also shown that the K_Ca_3.1 channel can regulates extracellular Ca^2+^ entry and thereby MSC proliferation.^11^ Further investigations are required to better understand how the K_Ca_3.1 channel is critically engaged in the transduction of mechanical forces to intracellular Ca^2+^ signalling and cell proliferation in MSCs.

In summary, this study shows a critical role of the K_Ca_3.1 channel in mediating mechanical stretch‐induced stimulation of BMSC proliferation, thus revealing a new molecular mechanism in the transduction of mechanical forces to MSC proliferation. Such a finding should be helpful in developing new strategies to maintain and stimulate the capacity of MSC proliferation and increase the cell number required for MSC‐based applications in regenerative medicine.

## CONFLICT OF INTEREST

The authors confirm that there are no conflicts of interest.

## AUTHOR CONTRIBUTIONS

YF developed and organized this paper. XJ, HS, XC, YH and YZ carried out experimental work. XJ and HS mainly drafted the paper and created the figures. PJ, CG, XG and YH assisted in data analysis and interpretation. LH J critically reviewed the manuscript. This manuscript is not under review elsewhere, and all authors read and approved the final manuscript.

## Data Availability

Data sets generated or analysed during the current study are included in the article.
