# Supplementary material for: A critical role of the KCa3.1 channel in mechanical stretch‐induced proliferation of rat bone marrow‐derived mesenchymal stem cells
Source: J Cell Mol Med. 2020 Feb 17;24(6):3739–44. doi: 10.1111/jcmm.15014 (PMC7131943; doi:10.1111/jcmm.15014)
Supplement: Supplementary file 1 [file JCMM-24-3739-s001.docx]

**Supplementary file**

**RT-PCR**

Total RNA was extracted using a Trizol RNA-prep kit (Qiagen) according to the manufacturer’s instructions. The RNA concentration and purity were determined using a Nanodrop 2000 spectrophotometer (Thermo Scientific). One µg RNA was reverse-transcribed into cDNA in a 25-µl reaction volume using a High Capacity RNA-to-cDNA kit (Applied Biosystems) and a Mastercycler Gradient PCR machine (Thermo Scientific) at 37ºC for 60 min. The reverse transcription was stopped at 95ºC for 5 min. One µl cDNA was used for PCR in a 10-µl reaction volume using a PCR kit (YTHX Biotechnology, China) and primers listed in the supplementary table 1. Glyceraldehyde 3-phosphate dehydrogenase (GAPDH) was used as an internal control. The PCR amplification was performed using the following conditions: 95°C for 1-5 min, 35 cycles (94°C for 15-30 s; 58°C for 30 s for K_Ca_3.1 and 57°C for GAPDH; 72°C for 20-30 s), and 72°C for 7 min. PCR products were separated on 2% agarose gels containing ethidium bromide and visualized with an UV transilluminator (Tanon, China). The intensity of PCR products was analyzed by Image J.

**Supplementary table 1 Primers used for PCR**

| **mRNA** | **Genebank**  **accession no.** | **Primer sequence (5’-3’)** | **Nucleotide**  **no.** | **Product**  **size (bp)** |
| --- | --- | --- | --- | --- |
| K_Ca_3.1 | NM023021 | Forward: GAACAAGTGAATTCCATGGT  Reverse: CTATGTGGCCTCCTGGATG | 1223-1417 | 195 |
| GAPDH | AF106860 | Forward: GTTGTCTCCTGTGACTTCA  Reverse: GGTGGTCCAGGGTTTCTTA | 1684-1867 | 184 |

**Electrophysiology**

Whole-cell patch-clamp recording of the K^+^ currents was performed using an EPC-10 amplifier and Patchmaster software (HEKA, Lambrecht, Germany). Briefly, borosilicate glass electrodes (1.2-mm outer diameter) were pulled with a Brown-Flaming puller (model P-97; Sutter Instrument, Novato, CA) and had tip resistance of ~ 5 MΩ when filled with pipette solution (in mM: 20 KCl, 110 K-aspartate, 1.0 MgCl_2_, 1.2 CaCl_2_, 10 HEPES, 0.05 EGTA, 0.1 GTP, 5.0 Na-phosphocreatine, and 5.0 Mg_2_ATP, pH 7.2). Cells were perfused with Tyrode solution (in mM: 136 NaCl, 5.4 KCl, 1.0 MgCl_2_, 1.8 CaCl_2_, 0.33 NaH_2_PO_4_, 10 glucose, and 10 HEPES, pH 7.3; 310 mOsm/kg H_2_O) or hypotonic solution, in which the NaCl concentration was reduced to 90 mM (223 mOsm/kg H_2_O). The duration of cells in hypotonic solution was less than 45 min. The membrane potential was held at -80 mV after whole-cell configuration was established. Currents were elicited by 500-ms step depolarization to potentials ranging from -60 mV to +60 mV with an increment of 10 mV. TRPM-34-sensitive currents were extracted from currents recorded before and after addition of 1 µM TRAM-34 in the same cells.


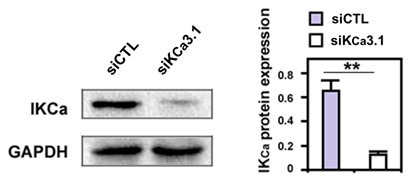


**A B**

**Fig. S1.** **Knockdown of the K_Ca_3.1 expression in BMSC using siRNA**. A, representative western blot showing the expression of K_Ca_3.1 and GAPDH in BMSC transfected with siK_Ca_3.1 or siCTL. B, summary of the mean K_Ca_3.1 expression as shown in panel A, from 3 independent experiments. Total proteins were harvested by RIPA buffer (Beyotime, Shanghai, China) adding 2% (v/v) phenylmethanesulfonyl fluoride (Sigma), quantified by the BCA kit (Beyotime). The proteins were separated by SDS-PAGE and blotted to PVDF membranes. After blocked using 5% non-fat milk for 1-2 h at room temperature, membranes were incubated with the primary anti-K_Ca_3.1 antibody (Alomone) overnight at 4°C and then with the secondary horseradish peroxidase-conjugated anti-mouse IgG antibody (Santa Cruz Biotechnology). Proteins were visualized using enhanced chemiluminescence (Applygen, Beijing, China) and images were captured by using MiniChemi (Sagecreation, China). GAPDH was used as the load control. The intensity of proteins was analyzed by Image J.
